# A scoping review to inform the development of dementia care competencies

**DOI:** 10.1177/14713012231165568

**Published:** 2023-03-28

**Authors:** Kelly Kay, Kateryna Metersky, Victoria Smye, Colleen McGrath, Karen Johnson, Arlene Astell, Winnie Sun, Emma Bartfay

**Affiliations:** Ontario Institute for Studies in Education, 7938 University of Toronto, Toronto, ON, Canada; Provincial Geriatrics Leadership Ontario, Toronto, ON, Canada; Daphne Cockwell School of Nursing, 7984 Toronto Metropolitan University, Toronto, ON, Canada; Arthur Labatt Family School of Nursing, 6221 Western University, London, ON, Canada; School of Occupational Therapy, 6221 Western University, London, ON, Canada; McCormick Dementia Services, London, ON, Canada; Department of Occupational Sciences and Occupational Therapy, 7938 University of Toronto, Toronto, ON, Canada; School of Psychology and Clinical Language Sciences, 6816 University of Reading, Reading, UK; DATE Lab, KITE Research Institute, University Health Network, Toronto, ON, Canada; Faculty of Health Sciences, 85458 Ontario Tech University, Oshawa, ON, Canada

**Keywords:** dementia, persons living with dementia, principles, competencies, education

## Abstract

Health professionals and care partners of persons living with dementia have expressed that learning needs related to dementia care are a priority. There are currently a variety of training programs available in Ontario (Canada) to address aspects of dementia care, but no commonly accepted description of the core knowledge, skills, and abilities, (i.e., competencies) that should underpin dementia-related training and education in the province. The aim of this study was to review current evidence to inform the later development of competency statements describing the knowledge, skills and actions required for dementia care among care providers ranging from laypersons to health professionals. We also sought to validate existing dementia care principles and align new concepts to provide a useful organizing framework for future competency development. We distinguished between micro-, meso- and macro-level concepts to clarify the competencies required by individuals situated in different locations across the healthcare system, linking competency development in dementia care to broader system transformation. This review precedes the co-development of a holistic competency framework to guide approaches to dementia care training in Ontario.

## Introduction

In Canada, approximately 25,000 individuals are diagnosed annually and nearly 690,000 Canadians are estimated to be living with dementia (Alzheimer Society Canada, 2016). Many Canadians living with dementia report difficulty accessing appropriate services and support (Alzheimer Society Canada, 2017). This challenge may be due, in part, to limited knowledge among some members of the public, healthcare professionals, and family and friend care partners about how to respond to, and best support, those living with dementia.

The National Dementia Strategy (Public Health Agency of Canada [PHAC], 2019) recommends building the capacity of care providers through increased access to, and adoption of, evidence-based and culturally appropriate guidelines for standards of care. PHAC (2019) defines care providers as family/friends, personal care workers, health professionals, and first responders (e.g. emergency medical services providers). This PHAC definition highlights the broad range of individuals and professions who require specific knowledge and skills to provide dementia care.

Learning needs related to dementia care were ranked among the top two categories of learning needs in a meta-summary of training needs assessments of Ontario health professionals (Lenard et al., 2015). This is supported by a finding that clinicians report that their core professional education does not adequately prepare them to provide dementia care (Lee at al., 2020). Similarly, Peterson et al. (2016) identified the need for public education to support care partners of persons living with dementia, to better understand symptoms and approaches to care, and to encourage their pursuit of accurate information in a timely manner.

Currently, there are a variety of forms of dementia education and training in Ontario, Canada. Examples include dementia webinars and care partner seminars offered by national, regional and local Alzheimer Societies and university-linked dementia education programs such as: McMaster University’s iGericare program (see https://igericare.healthhq.ca/en); the University of Waterloo’s Murray Alzheimer Research and Education Program (MAREP) (see https://the-ria.ca/programs/murray-alzheimer-research-education-program-marep/); and, the educational programs and tools of Lakehead University’s Rethink Dementia program (see http://www.rethinkingdementia.ca/). In addition, there are a number of health care worker-focused training programs including: P.I.E.C.E.S^™^ Canada and the Gentle Persuasive Approach(c) developed by AGE Inc.; Indigenous focused materials developed by the Indigenous Cognition and Aging Awareness Research Exchange (I-CAARE) (see https://www.i-caare.ca/); and courses offered in community colleges and workplaces as stand-alone courses or as part of a variety of dementia education certificates available for care partners and for healthcare workers. Each course or training program has its own curriculum, learning objectives and approach to content design, contributing to variation in dementia care practice and in the experiences of persons living with dementia who receive care from others.

The current educational offerings are important contributions to addressing knowledge gaps in dementia care. These educational opportunities also address learning needs across a broad range of audiences, from members of the lay public to health professionals. However, there is currently no commonly accepted description of the core knowledge, skills, and abilities, (i.e., competencies) that should underpin dementia-related training and education in Ontario, Canada.

Competency frameworks are used extensively in healthcare to coordinate health professional education and provide a systematic approach to the development, delivery, and evaluation of curricula. To our knowledge and through an extensive review of the literature we conducted, there is no comprehensive unified Ontario-wide framework that describes the competencies required for dementia care across all categories of carers.

Developing a competency framework for dementia care may assist in translating emerging dementia research findings into both lay and professional dementia education programs by helping to identify desired learning outcomes. Such a framework, particularly when co-developed with persons living with dementia and their care partners, could aid in aligning educational efforts with the identified aspirations and goals of individuals who live with dementia. Our aim was to review current evidence to inform the development of competency statements describing the knowledge, skills and actions required for dementia care among care providers ranging from laypersons to health professionals. We also sought to validate a preliminary adaptation of an existing dementia care competency framework (Bardsley, 2011), and, if feasible, align new concepts emerging from our review with established principles. This scoping review was designed as a preliminary step in a project planned to engage persons living with dementia, their care partners, and others in the development of a competency framework adapted for an Ontario context, which will be reported in a future manuscript.

### South West Dementia Partnership (United Kingdom)

Diane Bardsley (now Bardwell) was the Principal Program Lead, Dorset Clinical Commissioning Group, South West Dementia Partnership (UK) in 2011. Her competency framework (Bardsley, 2011) was selected and adapted as an analytic framework for this study because the principles underpinning her framework aligned closely with themes identified by two members of the research team (KK, VS). These themes arose during consultations conducted between 2014–2018 with persons living with dementia, care partners, and health professionals working in dementia care in Eastern Ontario and guided an early adaptation of Bardsley’s framework by the team (permission has been granted for adaptations).

Bardsley (2011) identified 13 principles to organize dementia care competencies within the UK context. These principles (Table 1) have been adapted with minor wording changes, for the Ontario, Canada context.

**Table 1.** Organizing principles for dementia care competencies (adapted from Bardsley, 2011).

a) Promote health and social well-being
b) Identify dementia - know the early signs
c) Assess and diagnose
d) Communicate sensitively/safely
e) Support living well with dementia; promote independence and activity
f) Understand and respond to unmet needs and signs of distress
g) Value and respect family and other care partners; support access to services
h) Work as part of a multi-agency team to provide support
i) Understand the context of care and support for persons living with dementia
j) Provide end of life care for persons living with dementia
k) Support for dementia worker personal development and self care
l) Accountability of leaders for processes and practices in dementia care
m) Quality improvement, accountability and evaluation is embedded in practice

These principles are used as an organizing framework to synthesize the findings of our review.

## Methods

The research team sought to identify a broad range of knowledge, skills, and actions relevant to dementia care that spanned the lay to professional continuum. With few existing examples of dementia care competency frameworks noted in a scan of the literature, a scoping review method was selected to gather the current state of the literature in dementia care competencies more broadly, beyond the few available, published competency frameworks. We used Arksey and O’Malley’s five-stage framework (2005) to guide the conduct of the review. The first stage in Arksey and O’Malley’s approach is identifying the research question. The following question was identified to guide this review – “What knowledge, skills and actions, and/or competency statements describe the expectations of members of the lay public and interprofessional health teams who provide service and care to persons living with dementia?” The search strategy used when undertaking this review included the use of a subject librarian and electronic databases.

The second stage revolves around identifying and selecting relevant studies. A 13-years paper publication date time frame was set (2008–2021) with the associated rationale being that significant changes continuously occur in the field and study of dementia care. This time frame reflects the study start date of 2018, and an additional, later, scan of the literature to ensure completeness and currency followed. The need was to retrieve competency statements that would assist with the creation of the most current, and up-to-date competency framework. In addition, the articles needed to be written in the English language and published in scientific, peer-reviewed journals.

The following electronic databases were searched: Cumulative Index to Nursing and Allied Health Literature (CINAHL), Medline, Scopus, and PsychINFO. All types of dementia were permitted in the search. The terms clinical competence, attitude, attitude of health personnel, judgement, competency/competencies, knowledge, and frameworks were searched in combination with the terms: caregiver, carer, family/ies, friend/s, spouse, healthcare/care provider, health professional, clinician, and staff (Diagram 1). For this search, the term ‘caregiver’ was included as it is more frequently used in the literature, despite the recent trend to prefer the term ‘care partner’ in everyday use. Our analysis reflects the term ‘caregiver’ to maintain consistency with the literature reviewed.

Diagram 1. Combination of Search Terms

1 Dementia/(46,888)
2 Alzheimer Disease/(87,111)
3 exp Dementia, Vascular/(6489)
4 Frontotemporal Lobar Degeneration/or Frontotemporal Dementia/(3326)
5 Lewy Body Disease/(2987)
6 dement*.tw. (100,196)
7 or/1–6 (171,740)
8 Clinical Competence/(85,855)
9 Health Knowledge, Attitudes, Practice/(100,604)
10 Attitude/or Attitude of health personnel/(160,481)
11 Judgment/(17,968)
12 (competency or competencies).tw. (26,845)
13 (knowledge* or skill* or attitude* or judg?ment*).ti,kf. (144,345)
14 (framework* or frame work*).tw. (223,174)
15 or/8–14 (643,459)
16 Home Nursing/(8827)
17 Family/(74,521)
18 Friends/(4449)
19 Spouses/(9233)
20 exp Health Personnel/(471,353)
21 exp Health Occupations/(1,655,230)
22 Patient Care/(9315)
23 Primary Health Care/(70,416)
24 (caregiver* or care giver* or carer* or family or families or friend or friends or spouse* or healthcare provider* or care provider* or health* professional* or clinician* or staff*).tw. (1,349,095)
25 or/16–24 (3,201,291)
26 7 and 15 and 25 (2753)
27 limit 26 to English language (2543)
28 remove duplicates from 27 (2336)

**Figure 1.**
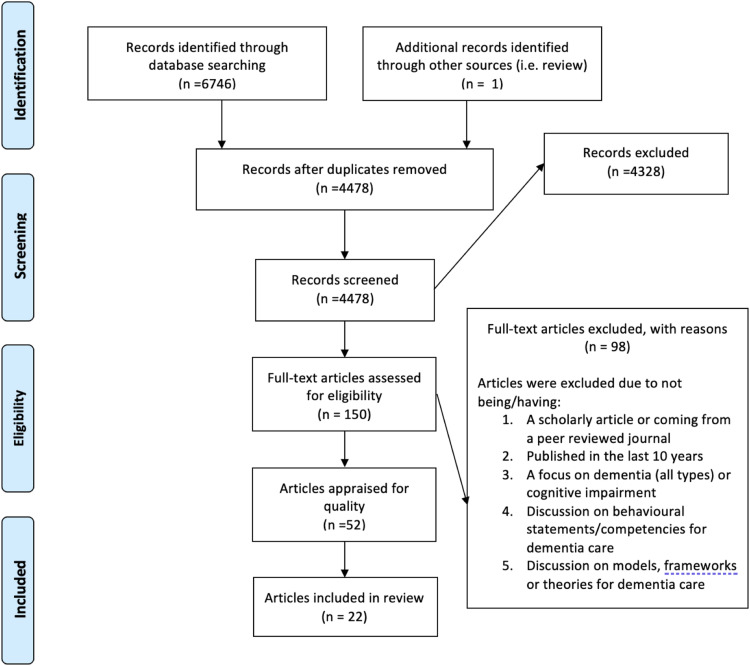
PRISMA chart.

A total of 6746 sources were retrieved using this search strategy and one additional record was identified during peer review; 2269 duplicates were removed, leaving 4478 sources for further review (Figure 1). The titles and/or abstracts of all 4478 sources were read independently by two reviewers to deem their relevance for inclusion in the review. Any conflicts in scoring were discussed and decisions were made on article inclusion. Sources that discussed any provider, methodology, behavioural statements, competencies, models/frameworks/theories, or dementia subtypes were included and moved forward to stage two: full text review. This resulted in a total of 150 sources proceeding to an independent full-text review by two researchers (e.g., members of the research team working in pairs with a subset of articles to review) to deem their suitability for inclusion and proceeding to article appraisal. The following excluded articles from the full text review: (1) conference abstract, letter to the editor, or opinion pieces; (2) published before 2008; (3) did not focus on dementia (all types) or cognitive impairments; (4) did not discuss behavioural statements for dementia care; and (5) did not discuss models, frameworks, or theories for dementia care.

The fourth stage of Arksey and O’Malley’s five-stage framework (2005) is charting the data. Prior to this, the team had decided to appraise the articles deemed relevant for inclusion in this review. Although in a scoping review, and the five-stage framework, there is no requirement for included studies to undergo quality appraisal, there is controversy about the need to assess methodological quality of studies. In fact, Bieber et al. (2019) found that nearly a quarter of studies published in 2014 included this step in their review. Thus, in this scoping review, and because of the above criteria, a total of 52 sources were deemed appropriate for the article appraisal stage. A two-researcher independent blind appraisal process took place using the following appraisal tools: AMSTAR Tool for Assessment of Methodological Quality of Systematic Reviews, Critical Appraisal Skills Programme (CASP) Qualitative Studies Appraisal tool, and the CASP Quantitative Studies Appraisal tool for RCTs. Of these 52, only 25 were deemed appropriate for inclusion as the other sources were either appraised as weak or moderate quality. Furthermore, three more articles were excluded as they focused on staff time given to supporting persons living with dementia, or attributes of the long-term care environment (e.g., staffing levels, management, leadership style) rather than competencies or behavioural statements relevant to dementia care. Thus, the final number of included articles was 22.

Only high-quality sources moved forward to the data extraction stage. A data extraction form was created to extract the following information from the included sources: full citation, location of where the study was carried out, aim/purpose of the study, setting, methodology, sample, whether persons living with dementia were included in the study, methods/data collection, data analysis methods, any information pertaining to framework for dementia care, results in relation to competency statements, strengths and limitations of the study, and relevance to the topic/research question and secondary question.

## Results

The final stage of the scoping review framework is collating, summarizing, and reporting the results (Arksey & O’Malley, 2005). The aim of this scoping review was to identify key concepts and competencies (i.e., knowledge, skills and actions) relevant to dementia care. We reviewed the characteristics of included studies and then summarized concepts and competencies, reflecting their alignment with our 13 organizing principles.

### Characteristics of Included Studies

Included studies reflected a heterogeneous sample of study designs or article types. Of the 22 included articles, three used quantitative designs, including a non-randomized control trial (Moyle et al., 2016), a cross-sectional survey design (Prentice et al., 2018), and a randomized trial of educational interventions (Conway & Chenery, 2016). Four studies used mixed methods (usually Delphi-style approaches with statistical evaluation) (Bond et al., 2016; Britten et al., 2018; Curyto & Vriesman, 2016; Isaksson et al., 2011). Six studies reflected various forms of systematic literature reviews (Carbonneau et al., 2010; Kolanoski et al., 2017; Prorok et al., 2013; Traynor et al., 2011; Tsaroucha et al., 2013; Van Mierlo et al., 2016). Eight were qualitative study designs that included grounded theory (DiLauro et al., 2017; Smeets et al., 2014; Smith et al., 2011), ethnographic-style studies (Caspi, 2015; Dowding et al., 2015; Scerri et al., 2015) and various forms of content analysis (De Vries et al., 2013; Robertshaw et al., 2019). One study used co-production and action research, and included a literature review, surveys, interviews, and focus groups (Carter et al., 2018).

**Figure 2.**
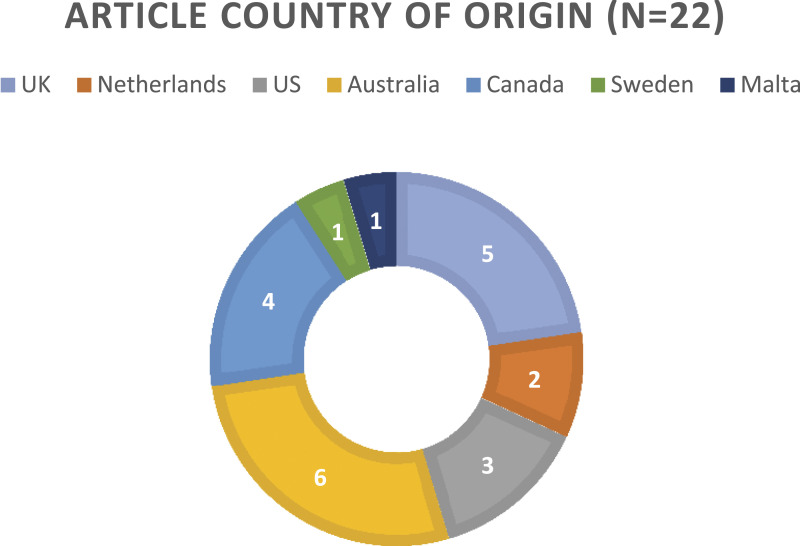
Articles and their country of origin.

The included studies were authored or conducted in seven different countries, with the most produced in Australia (Figure 2).

Seven studies reflected the long-term care setting and seven were relevant to the community setting (note: one article addressed both long term care and community). The remainder reflected the hospital environment, or were not setting-specific, meaning they did not describe specific environments (usually literature reviews).

Seven of the studies included either caregivers and/or persons living with dementia, of which three included caregivers only. The characteristics (e.g., country, aims, methodology, sample size, setting and major findings) of included studies are summarized (Table 2).

**Table 2.** Characteristics of included studies.

#	Author/Country	Aims	Methodology	Sample size/Setting	Major findings
1	Bond et al. (2016)/Australia	To develop expert consensus guidelines for how family and non-professional carers should assist a person who is developing cognitive impairment, or has dementia or delirium.	Delphi method: Expert panel; literature search; survey development	n = 65 (43 professional panel and 22 in carer advocate panel) Community	Development of a guideline document that explains what family and informal caregivers need to do and know when providing care to a person who is developing a cognitive impairment, has delirium or dementia.
2	Britten et al. (2018)/Australia	To develop gerontological nursing competencies for nurses for use across Australian nursing homes and community care.	Delphi method: 5 rounds of online consultation on core competencies and their associated domains	n = just over 400 participants. (Clinicians, managers, academics) Long term care Community	Development of 11 core competencies: Living well for older people across communities and groups; maximising health outcomes; communicate effectively; facilitating transitions in care; facilitating choices within legal and ethical frameworks; partnering with family carers, promoting mental health and psychological wellbeing; providing evidence-based dementia care; providing optimal pain management; providing palliative care; and enabling access to technology.
3	Carbonneau et al. (2010)/Canada	To develop a conceptual framework of the positive aspects of caregiving based on an integrative literature review.	Integrative literature review and conceptual framework development	n = 40 articles, theses and book chapters	Synthesis of existing literature into conceptual framework of positive aspects of caregiving for persons living with dementia.
4	Carter et al. (2018)/UK	To refresh the admiral nurse competency framework.	Co-production and action research Literature review Survey and interviews with admiral nurse practitioners Focus group with people living with dementia and families	n = 75 survey and 27 focus group participants (admiral nurses) n = 3 persons living with dementia who had received admiral nursing care n = 3 family members	Identified six overarching competencies: Person centred care; triadic relationship (person with dementia, carer and professional); best practice; sharing knowledge; and therapeutic skills. Also described expectations at levels of competency.
5	Caspi (2015)/USA	To identify the circumstances, sequence of events and triggers that lead to aggressive behaviors between residents, and to develop staff prevention strategies.	Qualitative- participant observations, informal conversations, review of clinical records, semi-structured interviews	n = 12 persons living with dementia and n = 13 staff and managers Long term care	Twelve effective staff prevention strategies were identified: being alert; being proactive; being informed about previous incidents in which a certain resident was involved; redirecting a resident from the area of where the aggressive behaviour occurred; offering the person to take a walk; separating; positioning, repositioning, or changing seating arrangements; switching or refocusing the topic or subject; distracting the person to a more pleasurable activity, diverting to a different activity or changing it; staying calm; not arguing with a resident who is displaying aggressive behaviour; seeking help from other staff.
6	Conway et al. (2016)/Australia	To evaluate the effects of a communication skills training program on community aged care staff’s knowledge of communication support in dementia and on staff’s care experiences.	A multi-centre-controlled pretest/post-test design with randomized cohort allocation	n = 38 (training group n = 22; control group n = 16) care staff working in community aged care. Community	Improved knowledge scores from baseline found for training group immediately post training (*p* = 0.002) and at 3-months follow up (*p* = 0.001). Training effect for self-efficacy (*p* = 0.024), strain in nursing care (0.023) and preparedness to provide care (*p* = 0.007). No significant difference for the control group on any measures. The training program was positively received by staff and had a significant effect on staff confidence and knowledge to care for persons living with dementia.
7	Curyto et al. (2016)/USA	To describe the development of the knowledge of dementia competencies self-assessment tool and report on its internal consistency and test-retest reliability. The tool was developed to help direct care workers (DCWs) assess their knowledge of 7 dementia competencies identified by the Michigan dementia coalition.	Tool development consisted of a literature search, expert panel consultation, DCW feedback and content validity ratings reflecting the relative importance of 7 dementia competencies	Item selection based on a literature review and expert panel consultation. Given to 159 DCWs and re-administered to 57 DCWs in a range of LTC settings. Long term care	Dementia competency areas included in order of importance: Care interactions (22%), understanding behaviours (18.5%), enriching the person’s life (13%), knowledge of dementia disorder (12.7%), person centred care (12.6%), interacting with families (11%) and DCW self-care (10.2%). 82 items in the final KDC-SAT. Good test re-test reliability (*p* < 0.001). Good internal consistency with Cronbach’s score of 0.906.
8	De Vries et al. (2013)/UK	To establish a set of competencies associated with the primary care liaison role in primary care.	Qualitative integrative review and consultation with key stakeholders and families and persons living with dementia.	Stakeholders n = 14, dementia patients and carers n = 70+, articles closely reviewed n = 400+ Community	There is a real need to increase timely diagnosis for persons living with dementia. There needs to be up-skilling to promote this skill. Competencies needed for timely diagnosis: (1) knowledge/awareness of dementia and dementia-related issues, (2) comprehensive understanding of the behaviours of individuals with MCI, dementia and other illness that have a neuropathology, (3) skilled in interacting with persons with a wide range of illnesses including MCI and dementia, (4) skilled in interacting with families and or significant others of persons at risk or with concerns about dementia, (5) competent at providing education/facilitation and giving information to families and caregivers, and (6) skilled in person-centered care approaches.
9	DiLauro et al. (2017)/Canada	To identify how caregivers perceive their spouses’ participation in leisure activities since dementia onset and the professional guidance caregivers require to increase persons' with dementia participation in shared leisure activities.	Exploratory qualitative descriptive; focus groups	n = 9 caregiver participants Community	Three major themes identified: Recognizing and acknowledging changes; making sense of changes and conflicts, embracing changes and forging ahead. Caregiver adaptation to change and engagement with joint shared leisure activities with people living with dementia is an important consideration in dementia care.
10	Dowding et al. (2015)/UK	To provide a revised conceptual model of decision making for the recognition, assessment and management of pain in patients with dementia in acute care settings.	Exploratory ethnographic qualitative, nested case study	n = 31 patients with dementia; n = 52 staff and n = 4 carers Hospital	A reconceptualized model of decision making related to pain assessment and management for patients with dementia was proposed (adapted from recognition-primed decision model). The revised model places emphasis on the salience of individual cognition and acknowledges that decisions are constructed through social interaction and organizational context.
11	Isaksson et al. (2011)/Sweden	To explore the types of caring situations, resident characteristics related to physically violent behaviours among residents with dementia and how professional caregivers managed these behaviours.	Mixed methods: Structured interviews using a questionnaire to assess residents' violent behaviour/residents' motor functions, vision, hearing, speech, ADL functions and psychiatric symptoms were measured with the multi-dimensional dementia assessment scale (MDDAS)	n = 309 residents Long term care	Physically violent behaviour is frequently displayed among residents in care homes and caregivers mainly manage these in a symptom-oriented way. Future interventions should aim to understand and manage these behaviours in residents.
12	Kolanowski et al. (2017)/USA	To provide a scoping review to seek what high quality evidence exists for patient, caregiver, environmental determinants regarding five specific behavioural and psychological symptoms of dementia (BPSD) (aggression, agitation, apathy, depression and psychosis).	Systematic review using an a priori review protocol	n = 56 high quality/low bias articles	Each symptom had its own set of determinants, but many were common across several symptoms: Neurodegeneration, type of dementia, severity of cognitive impairments, declining functional abilities, and caregiver burden and communication. The study provided context to understand and develop strategies for managing BPSD; provided guidance for leadership development in dementia care.
13	Moyle et al. (2016)/Australia	To assess (1) the effectiveness of the capabilities model of dementia care (CMDC) in assisting LTC staff to improve quality of life for older persons living with dementia living in LTC facilities; and (2) whether implementation of the CMDC improved staff attitudes towards, and experiences of, working and caring for the person with dementia.	A single blind, non-randomized controlled trial quantitative design	n = 81 staff Intervention group n = 51, control n = 30. n = 48 family members of persons living with dementia (intervention group n = 37; control n = 11) Long term care	LTC control group staff reported less work satisfaction (lower staff experiences of working with residents living with dementia questionnaire scores) compared to those in intervention group at 12 months. Family members in the control group reported lower levels of perceived quality of life for their family member with dementia when compared to the family members in the intervention group at 12 months. The CMDC appears to be an effective model of dementia care.
14	Prentice et al. (2018)/Canada	To identify self-perceived gaps in gerontological competencies among recreation staff in long-term care homes in Ontario, Canada.	Descriptive cross-sectional quantitative design	n = 487 recreation staff working in long-term care homes Long term care	Identified factors that recreational staff perceived as contributing to confidence in gerontological competencies: Experience, in-service training, education.
15	Prorok et al. (2013)/Canada	To conduct a systematic review of qualitative studies that examined aspects of health care experiences of persons living with dementia and their caregivers to better understand ways to improve care.	Systematic review of qualitative studies	n = 46 studies	Five major themes were identified: Seeking a diagnosis; accessing supports and services; addressing information needs; disease management; and communication and attitudes of healthcare providers. The healthcare experience was conceptualized as progressing through phases of seeking information and understanding, identifying the problem, role transitions following diagnosis and living with change.
16	Robertshaw et al. (2019)/UK	From the perspective of carers, families, healthcare professionals, and researchers, to create an understanding of views and experiences related to integrated dementia health and social care.	Qualitative - inductive open coding, framework analysis	n = 3058 online course learners and n = 847 course open online discussion board posts Community	Support for a model of integrated care, due to the holistic view of care that incorporates health and social care needs. The model should be person-centered and holistic, involving a multidisciplinary team of social and health care practitioners, the patient, family and wider community.
17	Scerri et al. (2015)/Malta	To explore quality dementia care from the point of view of formal care workers and family members of inpatients with dementia by asking them to remember and narrate the most positive care experiences in relation to caring for patients with dementia in the hospital. Factors that led to these positive experiences were also sought.	Exploratory qualitative design - appreciative inquiry framework	n = 33 staff of a geriatric hospital and n = 10 family members of persons living with dementia Hospital	The results from this study point to staff attributes, physical environment, organizational issues - staffing levels etc. and management (leadership style) as important factors in dementia care.
18	Smeets et al. (2014)/Netherlands	To explore factors that elucidate reasons for psychotropic drug prescription for neuropsychiatric symptoms in nursing home residents with dementia.	Qualitative with a grounded theory approach	n = 15 physicians and n = 14 nurses Long term care	Four themes with factors either or both limiting or enhancing psychotropic drug prescriptions were found and included: 1) mindset; 2) knowledge and experience; 3) communication and cooperation; and 4) external possibilities/limitations. A framework was developed to explain how different factors impact on psychotropic drug prescription.
19	Smith et al. (2011)/Australia	To describe the unmet needs of those with dementia living in remote communities of the Kimberley region, perceived by service providers and caregivers and to explore ways to facilitate improved care in this setting.	Qualitative – interviews and focus groups	n = 42 service providers and n = 31 caregivers and community-based care workers Community	Seven broad themes emerged: 1) caregiver role (subthemes: Reasons for being a caregiver; additional responsibilities; sharing caregiving role); 2) perspectives of dementia (causes of dementia; signs and symptoms); 3) community and culturally appropriate care (community engagement; community-based care; culturally appropriate activities); 4) workforce (Aboriginal staff; staff that are trusted and accepted; local support and guidance; accommodation; low pay and undervalued positions; domestic issues); 5) education and training (cultural training; dementia training; elder abuse training); 6) issues affecting remote communities (overcrowding; financial burden; transport); and 7) service issues (communication and coordination; intolerant attitudes; flexibility; distance of services from communities; interpreter use; clinical pathways and protocols; lack of services; specialist services; community case services; caregiver support services.
20	Traynor et al. (2011)/Australia	To review dementia nursing competencies, specific objective of explaining importance of said competencies across levels of practice and care settings.	Literature search and critical analysis	n = 59 publications; 86% grey lit, 14% academic	In relation to registered nurses and the health issue of dementia, there was determined to be a lack of clinical competency frameworks; however, this was also found with respect to frameworks for other healthcare workers. Some frameworks had been created, however, were limited to a specific group of practitioners. The need for various frameworks was identified. None reviewed were created across different levels of practice (applicable across a variety of workers).
21	Tsaroucha et al. (2013)/UK	To develop a set of generic core competencies to guide a competency-based curriculum in order to improve dementia training and education.	Systematic literature search	n = 18 literature frameworks	Development of eight core competencies and 87 knowledge, technical and attitude/behavioural skills statements in total.
22	Van Mierlo et al. (2016)/Netherlands	To develop an evidence-informed model (checklist) focusing specifically on factors that enable provision of individualized dementia care, to facilitate and promote community-based interventions.	Literature review of other studies conducted by the authors	NA	Model created that includes various implementations of personalized psychosocial care interventions that incorporate core components of personalized care. Provided a checklist for others who want to develop, implement, or evaluate personalized care interventions.

### Key Concepts and Competencies

The findings of the review were mapped to the 13 organizing principles described by Bardsley (2011). Several key concepts were identified and are summarized below that reflect the requisite competencies (e.g., knowledge, skills, and actions) required for effective dementia care.

#### a) Promote Health and Social Well-Being

A study by Bond et al. (2016) identified promoting mental health and psychological wellbeing as a required gerontological nursing competency. A second study by Tsaroucha et al. (2013) provided several specific suggestions to promote health and well-being through health promotion and dementia prevention strategies and promoting and empowering self-care activities. Further, competencies identified by Carter et al. (2018) included health promotion and prevention of dementia as relevant, although less frequently referenced in the literature.

#### b) Identify Dementia - Know the Early Signs

Five studies identified knowledge of dementia including causes, signs and symptoms (Bond et al., 2016; Curyto et al., 2016; De Vries et al., 2013; Smith et al., 2011; Tsaroucha et al., 2013) which are relevant to the early identification of dementia. Carter et al. (2018) also noted the need for the development of advanced competencies to facilitate the identification of rarer sub-types of dementia. Other studies noted that recognition and acknowledgement of changes (Di Lauro et al., 2017) and caregiver actions related to cognitive impairment, delirium, or dementia (Bond et al., 2016) were important concepts.

#### c) Assess and Diagnose

Four studies identified concepts related to the assessment and diagnosis of dementia, including providing evidence-based dementia care, and facilitating access to supports, services and disease management (Britten et al., 2018; Carter et al., 2018; Prorok et al., 2013; Tsaroucha et al., 2013). Additional competence in capacity assessment was also noted as relevant (Carter et al., 2018).

#### d) Communicate Sensitively/Safely

Five studies identified concepts relevant to communication, including communicating effectively and therapeutically (Britten et al., 2018; Carter et al., 2018; Tsaroucha et al., 2013), and demonstrating cultural competence reflecting the diverse needs of people living with dementia (Carter et al., 2018). In addition, attitudes that shape health professional communication (Prorok et al., 2013), and skills interacting with individuals, families or significant others about a variety of dementia related concerns (De Vries et al., 2013) were seen as essential aspects of dementia care.

#### e) Support Living Well with Dementia; Promote Independence and Activity

Eight studies included a variety of concepts related to supporting those who live with dementia to live well. Two studies identified macro or systems concepts such as living well across communities and groups, and facilitating choices within legal and ethical frameworks (Britten et al., 2018; Carter et al., 2018). Four studies referenced designing or implementing person centred care, including acquiring the necessary skills required to do so (Carter et al., 2018; Curyto et al., 2016; De Vries et al., 2013; Van Mierlo et al., 2016). Two studies identified optimizing health outcomes or experiences through a variety of approaches (e.g., supporting information seeking, role transition) (Britten et al., 2018; Prorok et al., 2013). The concept of embracing and living with change featured in DiLauro et al. (2017) and Prorok et al. (2013), worded eloquently in DiLauro et al. (2017) as “forging ahead”. Enriching the lives of persons living with dementia, including sharing leisure activities, and supporting individuals to engage in activities of interest featured in three studies (Curyto et al., 2016; Di Lauro et al., 2017; Tsaroucha et al., 2013).

#### f) Understand and Respond to Unmet Needs and Signs of Distress

Recognising factors influencing the use of psychotropic medications (e.g., mindset, knowledge, experience, communication, cooperation, external factors) was discussed as an important concept related to this principle in Smeets et al. (2014). Seven studies stressed the importance of understanding behaviours, including non-verbal communication (e.g., signs of hunger, boredom, frustration etc.) and using effective personal and environmental strategies to assist individuals with responsive behaviours (Carter et al., 2018; Caspi, 2015; Curyto et al., 2016; De Vries et al., 2013; Isaksson et al., 2011; Kolanowski et al., 2017; Tsaroucha et al., 2013). Three studies highlighted the importance of assessment and management of pain (Britten et al., 2018; Dowding et al, 2015; Tsaroucha et al., 2013). Broad consideration of the need to make sense of change and conflict, was also raised as a relevant concept by DiLauro et al. (2017).

#### g) Value and Respect Family and Other Caregivers; Support Access to Services

Partnering and interacting with caregivers in studies by Britten et al. (2018), Curyto et al. (2016), and Tsaroucha et al. (2013) and understanding of the caregiver role (e.g., reasons for being a caregiver, additional responsibilities, sharing the caregiver role) in Smith et al. (2011) were examples of concepts relevant to valuing and respecting family and caregivers. Smith et al. (2011) also noted the importance of providing culturally sensitive care, including the need for cultural training. In examining the literature, Carter et al., 2018 noted the requirement for skills to balance caregiver needs and those of individuals living with dementia featured prominently.

#### h) Work as Part of a Multi-Agency Team to Provide Support

Four studies identified concepts related to fostering models of integrated health and social care that include a variety of health professionals and specialists, and that may cross organizational boundaries (Robertshaw et al., 2019; Smith et al., 2011; Tsaroucha et al., 2013). According to Smith et al. (2011), knowledge and skills required by such teams include communication, coordination and case management, the ability to facilitate patient transitions, and the design and use of care pathways. Additionally, the ability to regard individuals, families, and care partners as not only the focus of support (Smith et al., 2011), but to enable their inclusion on the team (Robertshaw et al., 2019) were highlighted in two studies. Britten et al. (2018) highlighted the need for core competencies related to facilitating transitions in care, which necessitate support across agencies.

#### i) Understand the Context of Care and Support for Persons Living with Dementia

A range of relevant concepts related to the context of care were noted. Dementia care workers require knowledge and skill in engaging with communities to deliver community-based and culturally appropriate care, as per Smith et al. (2011) and Tsaroucha et al. (2013). Knowledge and skills in teaching and facilitation were also highlighted, to provide information to families and care partners (De Vries et al., 2013) and to help care partners identify positive aspects of caregiving for persons living with dementia (Carbonneau et al., 2010). Awareness and application of relevant legislation and dementia strategies were also important concepts related to the context of dementia care, as discussed in Tsaroucha et al. (2013).

#### j) Provide End of Life Care for Persons Living with Dementia

Two studies identified competencies related to the provision of palliative care as relevant gerontological nursing competencies (Britten et al., 2018; Carter et al., 2018). Supporting processes related to discussions with persons and care partners about end of life and advance care plans was also identified in Tsaroucha et al. (2013).

#### k) Support for Dementia Worker Personal Development and Self Care

Three studies noted the importance of staff education to increase worker knowledge, confidence and/or self-efficacy (Conway et al., 2016; Curyto et al., 2016; Prentice et al., 2018). Furthermore, the studies by Conway et al. (2016), Curyto et al. (2016), and Tsaroucha et al. (2013) highlighted the importance of supporting self-care among dementia workers, including managing stress, grief, loss, and personal safety, among other aspects of self-care. Specific training topics might include cultural training, dementia care, gerontological care, and elder abuse training, as discussed in Prentice et al. (2018) and Smith et al. (2011). Knowledge sharing, promoting best practices, and engaging in reflective practice were highlighted among overarching competencies expected of advanced practice nurses (Carter et al., 2018).

#### l) Accountability of Leaders for Processes and Practices in Dementia Care

Several studies highlighted the knowledge, skills, and actions required of leaders to enable effective processes and practices in dementia care. Four studies identified leadership actions relevant to macro or meso infrastructure development, such as enabling access to technology (Britten et al, 2018), ensuring the provision of interpretation services (Smith et al., 2011) and providing leadership in addressing environmental determinants of responsive behaviours (Kolanowski et al., 2017; Scerri et al., 2015). Five studies highlighted the role of leaders in implementing care models, as well as organizational leadership and management strategies that can best support dementia care, including those that foster collaboration across professional and organizational boundaries (Kolanowski et al., 2017; Moyle et al., 2016; Scerri et al., 2015; Traynor et al., 2011; Tsaroucha et al., 2013). Studies by Scerri et al. (2015) and Smith et al. (2011) highlighted concepts related to human resources competencies. These studies highlighted service planning, including rural/remote health considerations (Smith et al., 2011) and managing the workforce and balancing demands (Carter et al., 2018; Scerri et al., 2015; Smith et al., 2011) as core concepts. Examples of required knowledge and skills include attending to staff attitudes and attributes (Scerri et al., 2015; Smith et al., 2011) and addressing workforce development, including remuneration and the perceived value of the dementia care workers (Smith et al., 2011). The study by Smith et al. (2011) further noted the need for particular focus on development of the Indigenous dementia care workforce.

#### m) Quality Improvement, Accountability and Evaluation is Embedded in Practice

Two studies offered insights relevant to evaluation in dementia care and identified several desired outcomes for persons living with dementia (Moyle et al., 2016) and/or their caregivers (Carbonneau et al., 2010). In these two studies, the capabilities model described by Moyle et al. (2016) and the conceptual framework described by Carbonneau et al. (2010), detailed outcome statements were noted and are summarized (Table 3).

**Table 3.** Desired outcomes for persons living with dementia and their caregivers.

Desired outcomes for persons living with dementia	Desired outcomes for caregivers
Model/Framework	
Capabilities model of dementia care (Moyle et al., 2016: p. 1092, p. 1092)	Conceptual framework of positive aspects of caregiving for persons living with dementia (Carbonneau et al., 2010: p. 330, p. 330)
- “Feel valued”	- “Caregiver’s sense of self-efficacy” (determining factors)
- “Experience the best health possible”	- “Enrichment events in daily life” (determining factors)
- “Live independently with compassionate support from important others”	- “Meaning of the caregiver’s role in daily life” (domains of positive aspects of caregiving)
- “Enjoy pleasurable experiences through senses, imagination, and thought”	- “Quality of caregiver/care receiver daily relationship” (domains of positive aspects of caregiving)
- “Experience and express emotion in a way that is true to oneself”	- “Caregiver’s feeling of accomplishment” (domains of positive aspects of caregiving)
- “Reflect and decide on things that matter to oneself, including plans for the future and end of life”	- “Caregiver’s well-being and involvement continuity” (outcome)
- “Experience connection with others where one can contribute and there is self-respect, dignity, and a sense of shared humanity with individuals and the wider community”	
- “Live in a way where engaging with nature is a natural part of life”	
- “Play in a way that is meaningful and fun for oneself”	
- “Experience a sense of control over how one lives one’s life”	

Our review demonstrates good alignment between the current literature and prior work identifying principles for holistic dementia care. The results are summarized by principle (Table 4).

**Table 4.** Alignment of literature reviewed by dementia care principles.

	Reference	Principles (adapted with permission from Bardsley, 2011)												
		a) Promote health and social well-being	b) Identify dementia - know the early signs	c) Assess and diagnose	d) Communicate sensitively and safely	e) Support living well with dementia: Promote independence and activity	f) Understand and respond to unmet needs and signs of distress	g) Value and respect family and other caregivers: Support access to services	h) Work as part of a multi-agency team to provicde support	i) Understand the context of care and support for people living with dementia	j) Provide end of life care for people living with dementia	k) Support for dementia work personal development	l) Accountability of leaders for processes and practices in dementia care	m) Quality improvement, accountability and evaluation is embedded throughout practice
1	Bond et al. (2016)		X											
2	Britten et al. (2018)	X		X	X	X	X	X	X		X		X	
3	Carbonneau et al. (2010)									X				X
4	Carter et al. (2018)	X	X	X	X	X	X	X			X	X	X	
5	Caspi (2015)						X							
6	Conway et al. (2016)											X		
7	Curyto et al. (2016)		X			X	X	X				X		
8	De Vries et al. (2013)		X		X	X	X			X				
9	DiLauro et al. (2017)		X			X	X							
10	Dowding et al. (2015)						X							
11	Isaksson et al. (2011)						X							
12	Kolanowski et al. (2017)						X						X	
13	Moyle et al. (2016)												X	X
14	Prentice et al. (2018)											X		
15	Prorok et al. (2013)			X	X	X								
16	Robertshaw et al. (2019)								X					
17	Scerri et al. (2015)												X	
18	Smeets et al. (2014)						X							
19	Smith et al. (2011)		X					X	X	X		X	X	
20	Traynor et al. (2011)												X	
21	Tsaroucha et al. (2013)	X	X	X	X	X	X	X	X	X	X	X	X	
22	Van Mierlo et al. (2016)					X								
	Total references	3	7	4	5	8	11	5	4	4	3	6	8	2

Of particular note is the comprehensive work of Tsaroucha et al., (2013), which identified 87 specific “skills” (comparable to what we might call behavioural or competency statements). This comprehensive work, along with the additional insights offered by other authors, can be further mined to support the development of competency statements that can be included in a fulsome framework for dementia care in Ontario.

## Discussion

Through our scoping review, we noted a robust body of literature that can inform competency development in several specific areas (e.g., Support Living Well with Dementia: Promote Independence and Activity, Understand and Respond to Unmet Needs and Signs of Distress). In other instances, the current literature is limited (e.g., Promote Health and Social Well-being; Quality Improvement, Accountability and Evaluation). There were only a few references specific to providing culturally sensitive care (e.g., two references) and none addressing the needs of sub-populations of individuals living with dementia (e.g., people living with young onset dementia). These gaps in literature suggest areas for development and further study of the knowledge, skills, and actions (i.e., competencies) required by a range of actors (e.g., lay public, health professionals, persons living with dementia etc.) to address the broad spectrum of needs and experiences in dementia care.

The findings also suggest micro-,meso-and macro-level concepts that can shape the development of an overarching competency framework in dementia care. Micro-level concepts are those that could be carried out by individual actors, such as members of the lay public, family or friend caregivers, unregulated care providers and/or regulated health professionals. Such concepts include: communication skills; facilitation skills; knowledge about the signs and symptoms of dementia; partnering with persons living with dementia and care partners; and, supporting individuals experiencing responsive behaviours. Meso-and macro-level concepts are those expected of leaders and organizations and include concepts such as infrastructure, care models and workforce development. The distinction between micro-,meso-and macro-level concepts links to other work describing integrated care (Horgan et al., 2020) and helps to clarify the competencies required by individuals situated in different locations across the healthcare system. This understanding links competency development in dementia care to broader system transformation, where there is the potential to embed high quality dementia care more concretely across the health care system.

All concepts identified during this review could be mapped to the 13 organizing principles drawn from Bardsley (2011). This reinforces the comprehensiveness of earlier work, and our recent adaptation of it, and provides a useful organizing framework for future competency development.

The sixth optional stage of the Arksey and O’Malley’s (2005) scoping review framework is consultation. Consultation from key stakeholders was sought as part of this review and accomplished by expanding our research team to include individuals working in dementia care. We will also utilize the findings of this review to focus the planned co-development of the final competency framework with persons living with dementia, care partners and health professionals. It is anticipated this future work will develop, in detail, the expected competencies that span the range of individuals involved in caring for those impacted by dementia.

## Limitations

Our initial search yielded 6746 articles, a volume that was somewhat challenging for our research team to review. More stringent search criteria may have eliminated the volume of articles returned but may have also caused us to miss some of those studies that provided valuable insights. We elected to err on the side of inclusivity as competency-based approaches to dementia care is an emerging area of study.

## Conclusions

This scoping review was conducted to identify current literature relevant to the development of a competency-based framework for dementia care that spans the caring continuum, from members of the lay public to healthcare professionals. We identified 22 articles and confirmed 13 foundational principles that can serve to focus future competency development and inform curriculum design in dementia care training for the lay public, persons living with dementia, care partners and health care professionals. There are also implications at meso and macro levels for health care leaders and policy makers related to potential enablers for effective dementia care, which can be a focus for future study.

We anticipate using the results of this scoping review to inform the co-development of a holistic competency framework to guide approaches to dementia care training that is squarely focused on what matters most to persons living with dementia. We also anticipate this review will be of relevance to those interested in dementia care education across Ontario and in other jurisdictions.
